# The Effect of pH on Antibiotic Efficacy against *Coxiella burnetii* in Axenic Media

**DOI:** 10.1038/s41598-019-54556-6

**Published:** 2019-12-02

**Authors:** Cody B. Smith, Charles Evavold, Gilbert J. Kersh

**Affiliations:** 1 Rickettsial Zoonoses Branch, Centers for Disease Control and Prevention, Atlanta, GA 30329 USA; 2 Present Address: Program in Immunology, Harvard Medical School, Boston, MA 02115 USA

**Keywords:** Bacterial infection, Antibiotics

## Abstract

*Coxiella burnetii*, the etiologic agent of Q fever, replicates in an intracellular phagolysosome with pH between 4 and 5. The impact of this low pH environment on antimicrobial treatment is not well understood. An *in vitro* system for testing antibiotic susceptibility of *C. burnetii* in axenic media was set up to evaluate the impact of pH on *C. burnetii* growth and survival in the presence and absence of antimicrobial agents. The data show that *C. burnetii* does not grow in axenic media at pH 6.0 or higher, but the organisms remain viable. At pH of 4.75, 5.25, and 5.75 moxifloxacin, doxycycline, and rifampin are effective at preventing growth of *C. burnetii* in axenic media, with moxifloxacin and doxycycline being bacteriostatic and rifampin having bactericidal activity. The efficacy of doxycycline and moxifloxacin improved at higher pH, whereas rifampin activity was pH independent. Hydroxychloroquine is thought to inhibit growth of *C. burnetii in vivo* by raising the pH of typically acidic intracellular compartments. It had no direct bactericidal or bacteriostatic activity on *C. burnetii* in axenic media, suggesting that raising pH of acidic intracellular compartments is its primary mechanism of action *in vivo*. The data suggest that doxycycline and hydroxychloroquine are primarily independent bacteriostatic agents.

## Introduction

*Coxiella burnetii* is a gram-negative bacterium that is the etiologic agent of the human disease Q fever. This bacterium has a reduced genome and therefore requires infection and establishment of an intracellular niche for replication. *C. burnetii* replicates in mammalian host cells within the low pH environment of the phagolysosome^1^. *C. burnetii* can infect a wide variety of vertebrates, including mammals, reptiles, amphibians, and birds^2^. The most common reservoirs associated with human infection are domesticated farm animals and livestock, such as goats, sheep, and cows^2^. *C. burnetii* can be released into the environment in large numbers by shedding from infected animals in the placenta, feces, urine, or milk. *C. burnetii* has a very low infectious dose, with as few as 1–10 organisms capable of initiating an infection^3^. Q fever is most commonly spread via inhalation of aerosols that contain the bacteria. Q fever is present in most parts of the world.

*C. burnetii* has two distinct morphological forms, a small cell variant (SCV) and a large cell variant (LCV) which exist in a biphasic development cycle^4^. The SCVs are 0.2 to 0.5 μm in length, while the LCVs are more than 1 μm in length^4^. *C. burnetii* in the LCV stage are metabolically and reproductively active and found in the phagolysosome^5^. As SCVs, *C. burnetii* have reduced metabolic activity and exhibit spore-like characteristics, such as resistance to desiccation, ultraviolent light, and high temperatures. Much like bona fide bacterial spores, the SCV can remain viable in the environment for long periods of time, potentially over a year^6^. Upon infection, SCVs undergo morphological changes and convert into LCVs as they replicate exponentially before eventual transition back to SCVs^5^.

Q fever manifests in two forms, acute and chronic disease. Acute Q fever is characterized by a self-limiting febrile illness, often associated with flu-like symptoms including fever, headache, chills, and myalgia. More severe symptoms can include hepatitis and pneumonia, and may result in hospitalization. Chronic Q fever presents most commonly as either culture negative endocarditis or vascular infection. It is estimated that 2–5% of *C. burnetii* infections will develop into chronic Q fever^7^. It may take many months, if not years after the initial *C. burnetii* infection for chronic Q fever to become apparent. Patients with pre-existing valvulopathies or vascular disease are more at risk for development of chronic Q fever^8^. These chronic infections are difficult to treat, and are typically fatal if untreated^9^.

Antibiotic therapies are recommended for the treatment of Q fever. For acute Q fever, 100 mg doxycycline twice a day for two weeks is the advocated treatment^10^. Chronic Q fever, conversely, is much more difficult to treat. A 100 mg dose of doxycycline twice daily and 200 mg hydroxychloroquine three times a day is recommended. Therapy should continue for a minimum of 18 months, and may be required to continue for years^10^. Alternative treatments include fluoroquinolones, macrolides, trimethoprim/sulfamethoxazole, and rifampin^11^, but alternative treatments have been associated with greater risk of relapse and longer treatment times compared to the recommended treatment of doxycycline plus hydroxychloroquine^12^. It has been suggested that the ability of hydroxychloroquine to increase the pH of the phagolysosomal compartments where *C. burnetii* replicates increases the bactericidal activity of doxycycline and thereby makes the combination therapy more effective^13,14^, but a mechanism by which higher pH increases bactericidal activity has not been proposed.

There are several challenges in the treatment of chronic Q fever. Even with proper treatment, some cases can persist for more than 5 years^15^. Compliance is difficult for such a long therapeutic regimen, and incomplete clearance followed by a return of symptoms is not uncommon^7^. There are also many adverse side effects associated with long term use of doxycycline, including loss of appetite, nausea and vomiting, diarrhea, retinopathy, and sensitivity to sun exposure^16^.

*C. burnetii* can be grown in media without the need for host cell infection. Acidified citrate cysteine media (ACCM-2) is an axenic media which replicates the conditions within the phagolysosome, supporting growth of *C. burnetii* without the variables associated with host cell lines^17^. Limited studies have been done with antibiotic treatments in ACCM-2^18^. *C. burnetii* is metabolically active at a pH between 4 and 5, which is naturally found within the phagolysosome^19^. The impact of this low pH environment on antibiotic treatment of *C. burnetii* is not well understood. Use of ACCM-2 allows the pH of the *C. burnetii* environment to be manipulated, thus allowing direct testing of the effects of pH on the life cycle of *C. burnetii*. The aim of this study is to investigate the role that pH plays on the efficacy of antibiotics against *C. burnetii*.

## Results

### pH dependence of *C. burnetii* growth in axenic media

**Figure 1:**
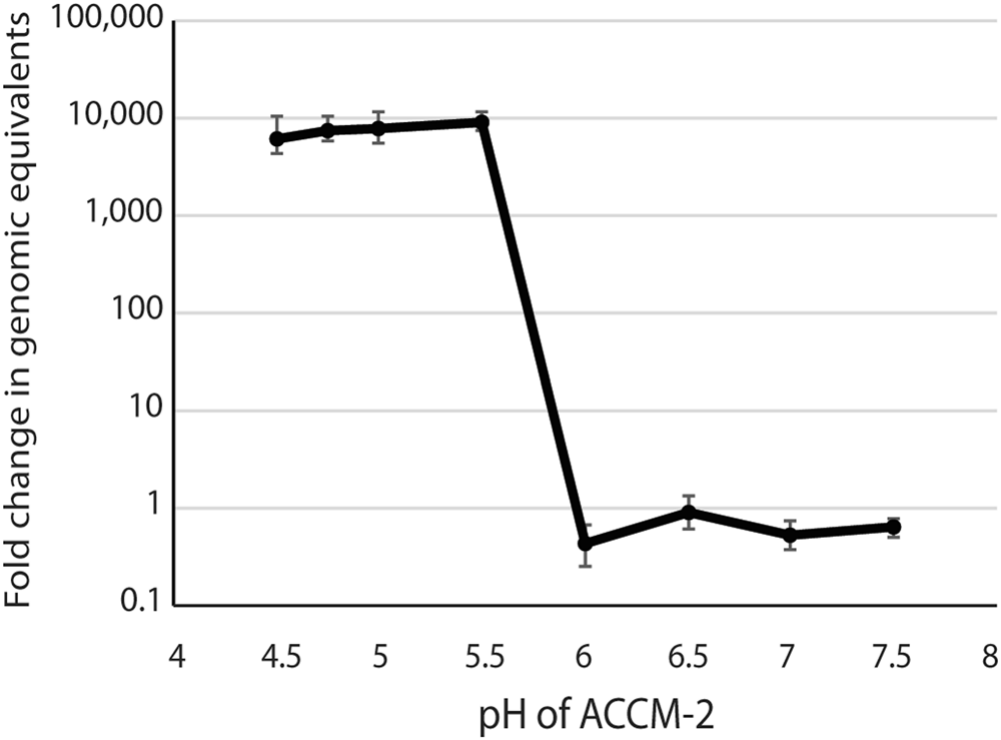
Growth of *Coxiella burnetii* Nine Mile Phase 2 in ACCM-2 medium. Genomes in the culture were calculated at the start of the culture and again on day 8 using qPCR. Robust growth of *C. burnetii* was observed at pH 5.5 and lower, but not at pH 6 and higher. Error bars represent 95% confidence intervals.

To establish the impact of pH on the ability of *C. burnetii* to replicate in ACCM-2 media, growth of *C. burnetii* Nine Mile Phase 2 (NMP2) was measured in ACCM-2 liquid culture at pH ranging from 4.5 to 7.5. The greatest expansion was observed at pH 5.5 with a 7-day fold change in NMP2 genomic equivalents of 9,040. The fold change in NMP2 genomic equivalents decreased with increasing acidity of the media with 7675, 7562, and 6127 fold expansion at pH 5, 4.75, and 4.5, respectively. No growth was detected at pH 6.0, 6.5, 7.0, and 7.5 (Fig. 1). The lack of growth at pH 6 and higher correlates well with a previous report of reduced *C. burnetii* metabolic activity at pH 6 and above^19^.

### Antibiotic susceptibility of *C. burnetii* in axenic media

To establish an assay for *C. burnetii* antibiotic susceptibility in ACCM-2 media, a panel of antibiotics were tested for the ability to prevent growth of *C. burnetii*. In this assay, cultures at day 4 of growth were treated with antibiotics. As previously demonstrated, use of *C. burnetii* at day 4 of growth in ACCM-2 ensured that the exposed organisms were almost all in the LCV form and metabolically active^20^. The use of already actively replicating organisms reduced the amount growth in untreated cultures compared to Fig. 1. With the exception of rifampin, antibiotics were tested at 10 μg/ml, which is above the peak serum concentration (Cmax) for all of these antibiotics used at standard dosing^16^. Rifampin was an exception because it has a higher Cmax and was therefore used at 20 μg/ml. In the absence of antibiotics, NMP2 expanded 63-fold in these cultures (Table 1). Doxycycline, rifampin, moxifloxacin, levofloxacin, and ciprofloxacin were very effective at preventing *C. burnetii* growth in this system with fold expansion between 0.05 and 0.11 (Table 1). In contrast, the macrolides azithromycin and erythromycin had very little impact on *C. burnetii* growth in ACCM-2 with fold changes of 53.2 and 58.6, respectively (Table 1).

**Table 1:** Efficacy of antibiotics against *C. burnetii* in ACCM-2 culture.

Antibiotic	Concentration (μg/ml)	Fold change (95% CI)	Efficacy
untreated	—	62.9 (46.7–83.1)	−
Doxycycline	10.0	0.09 (0.07–0.11)	+
Rifampin	20.0	0.11 (0.09–0.13)	+
Azithromycin	10.0	53.2 (37.1–70.6)	−
Erythromycin	10.0	58.6 (47.6–70.7)	−
Moxifloxacin	10.0	0.06 (0.05–0.13)	+
Levofloxacin	10.0	0.09 (0.05–0.13)	+
Ciprofloxacin	10.0	0.05 (0.05–0.06)	+

### Antibiotic efficacy with increasing pH

**Figure 2:**
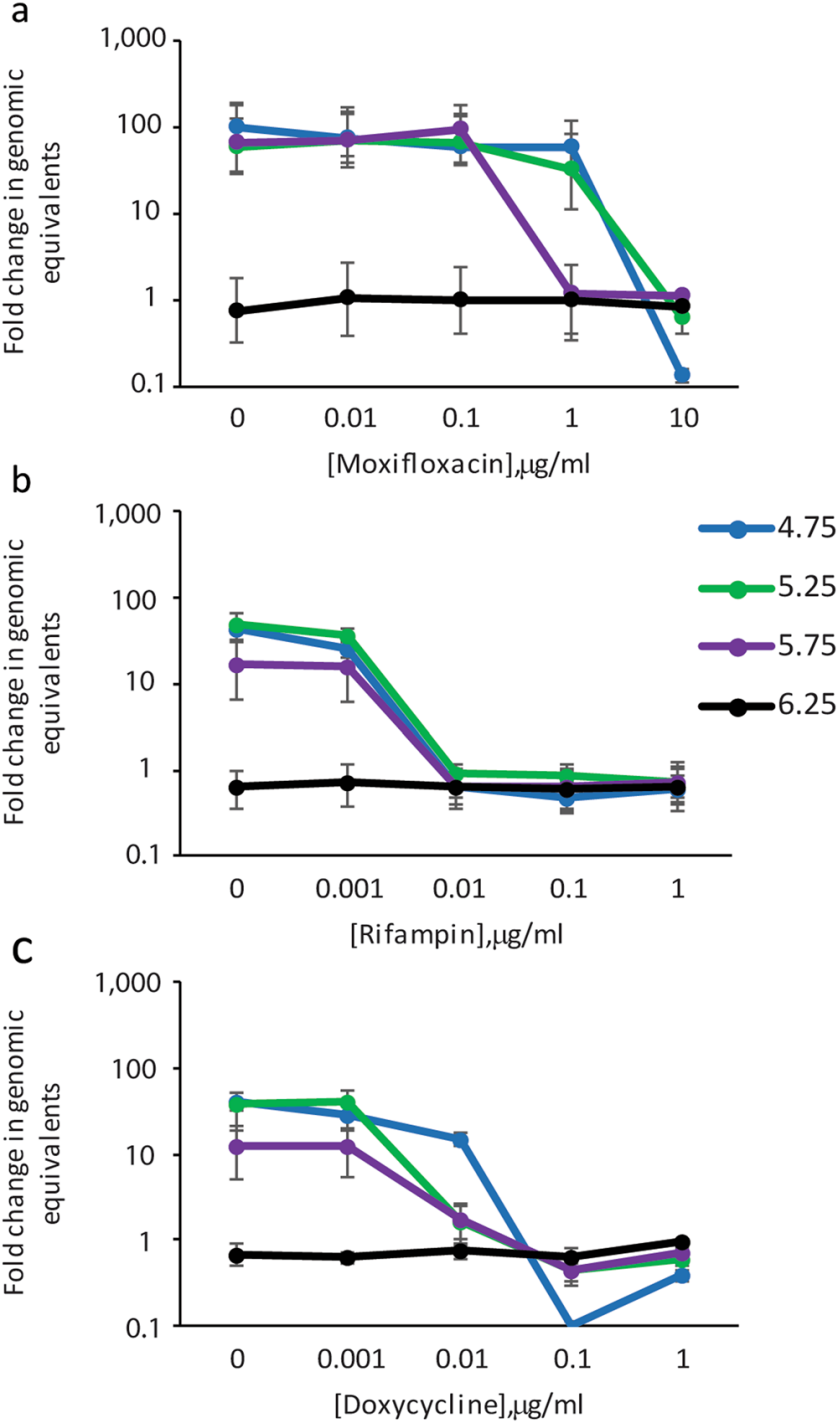
*C. burnetii* growth in ACCM-2 in the presence of moxifloxacin (**a**), rifampin (**b**), and doxycycline (**c**) at varying pH. Genomes in the culture were calculated at the start of the culture and again on day 7 using qPCR. The ratios of the means of genomes at day 7 to the means of genomes at the beginning of the culture were calculated to determine fold change in genomic equivalents. The standard errors of the means of genome equivalents were calculated and used to determine the upper and lower bounds of the ratios (fold change). The error bars depict the upper and lower bounds of fold change for each condition.

To compare efficacy of three different classes of antibiotics against *C. burnetii* in ACCM-2 and to evaluate the role of pH in antibiotic efficacy, NMP2 was exposed to doses of doxycycline, rifampin, and moxifloxacin at a variety of pH using actively growing cultures of NMP2. Without antibiotics added, the NMP2 expanded 40–100 fold at optimum pH of 4.75, and had no growth at all at pH 6.25 (Fig. 2). All three antibiotics were effective at preventing growth at pH 4.75, 5.25, and 5.75 (Fig. 2). Rifampin and doxycycline had similar potency in this system, with a minimum inhibitory concentration (MIC) of 0.01 μg/ml at pH 4.75, 5.25, and 5.75. Moxifloxacin was less potent and had an MIC of 10 μg/ml at pH 4.75 and 5.25, and an MIC of 1 μg/ml at pH 5.75.

**Figure 3:**
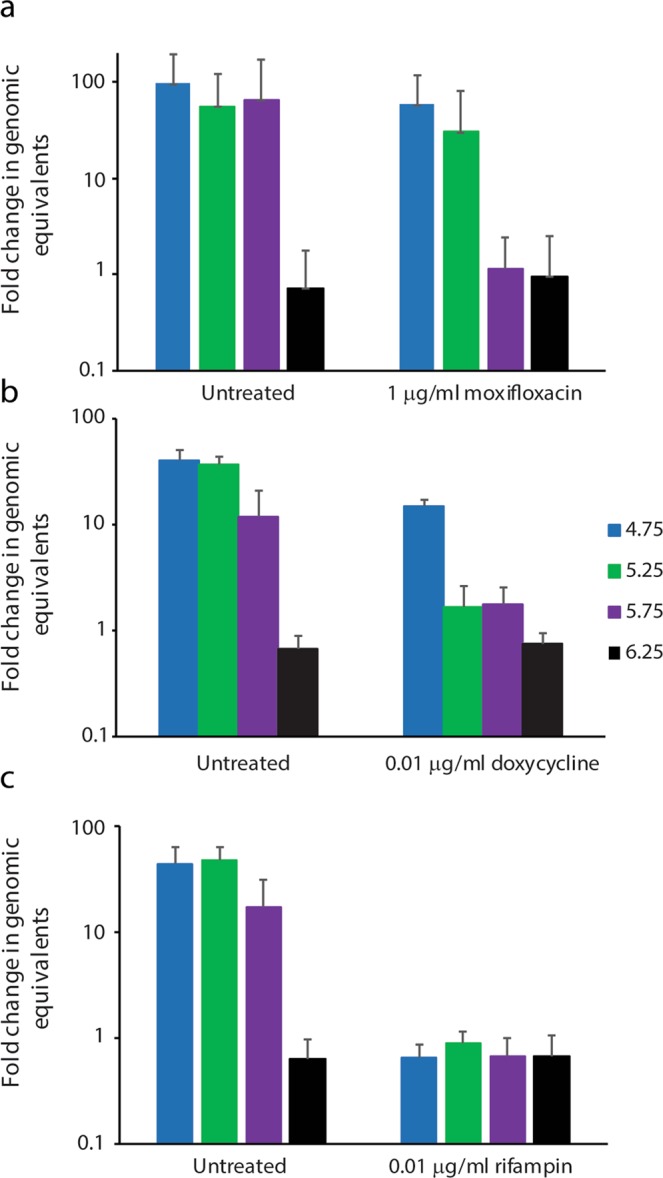
Moxifloxacin and doxycycline are less effective at reducing growth of *C. burnetii* at more acidic pH. Genomes in the culture were calculated at the start of the culture and again on day 7 using qPCR. The ratios of the means of genomes at day 7 to the means of genomes at the beginning of the culture were calculated to determine fold change in genomic equivalents. The standard errors of the means of genome equivalents were calculated and used to determine the upper and lower bounds of the ratios (fold change). The error bars depict the upper and lower bounds of fold change for each condition.

The higher MICs for moxifloxacin at pH 4.75 and 5.25 suggests that pH may play a role in efficacy of antibiotics. This is better visualized in Fig. 3 where the same fold change data for NMP2 is displayed in untreated versus antibiotic treatment at a concentration near the MIC for the three antibiotics. Susceptibility of NMP2 to rifampin is unaffected by pH changes, whereas doxycycline is slightly less effective at pH 4.75 and moxifloxacin has reduced efficacy at pH 4.75 and 5.25 (Fig. 3).

### Viability after antibiotic treatment

To determine if there was any bactericidal effect of these antibiotics in ACCM-2, at the end of the 7 day culture NMP2 was plated on ACCM-2 agar plates without antibiotics. Growth was evaluated after 14 days incubation on the agar plates. For moxifloxacin and doxycycline, growth on the agar plate was observed no matter what concentration of antibiotic was used in the 7 day culture (Table 2), indicating that these drugs have only bacteriostatic activity in this system. In a separate experiment, doxycycline concentrations of 5 and 10 μg/ml were also bacteriostatic for NMP2 (data not shown). For rifampin, no growth on agar plates was detected at pH 4.75 and 5.25 after culture with dosages of 0.01, 0.1, and 1 μg/ml, as well as pH 5.75 after culture with dosages of 0.01 and 1.0 μg/ml (Table 2). This demonstrates bactericidal activity against *C. burnetii* for rifampin in this system. The data also show that the lack of *C. burnetii* growth at pH 6.25 and above is a bacteriostatic effect and not due to killing of *C. burnetii*.

**Table 2:** Viability of *C. burnetii* after one week culture in ACCM-2 with the indicated antibiotics. A “+” indicates growth on ACCM-2 plates after dilution from the treated culture. A “-” indicates no growth.

pH	[moxifloxacin], μg/ml				
	0	0.01	0.1	1	10
4.75	+	+	+	+	+
5.25	+	+	+	+	+
5.75	+	+	+	+	+
6.25	+	+	+	+	+
	**[doxycycline], μg/ml**				
**pH**	**0**	**0.001**	**0.01**	**0.1**	**1**
4.75	+	+	+	+	+
5.25	+	+	+	+	+
5.75	+	+	+	+	+
6.25	+	+	+	+	+
	**[rifampin], μg/ml**				
**pH**	**0**	**0.001**	**0.01**	**0.1**	**1**
4.75	+	+	−	−	−
5.25	+	+	−	−	−
5.75	+	+	−	+	−
6.25	+	+	+	+	+

### Effect of hydroxychloroquine in axenic media

**Figure 4:**
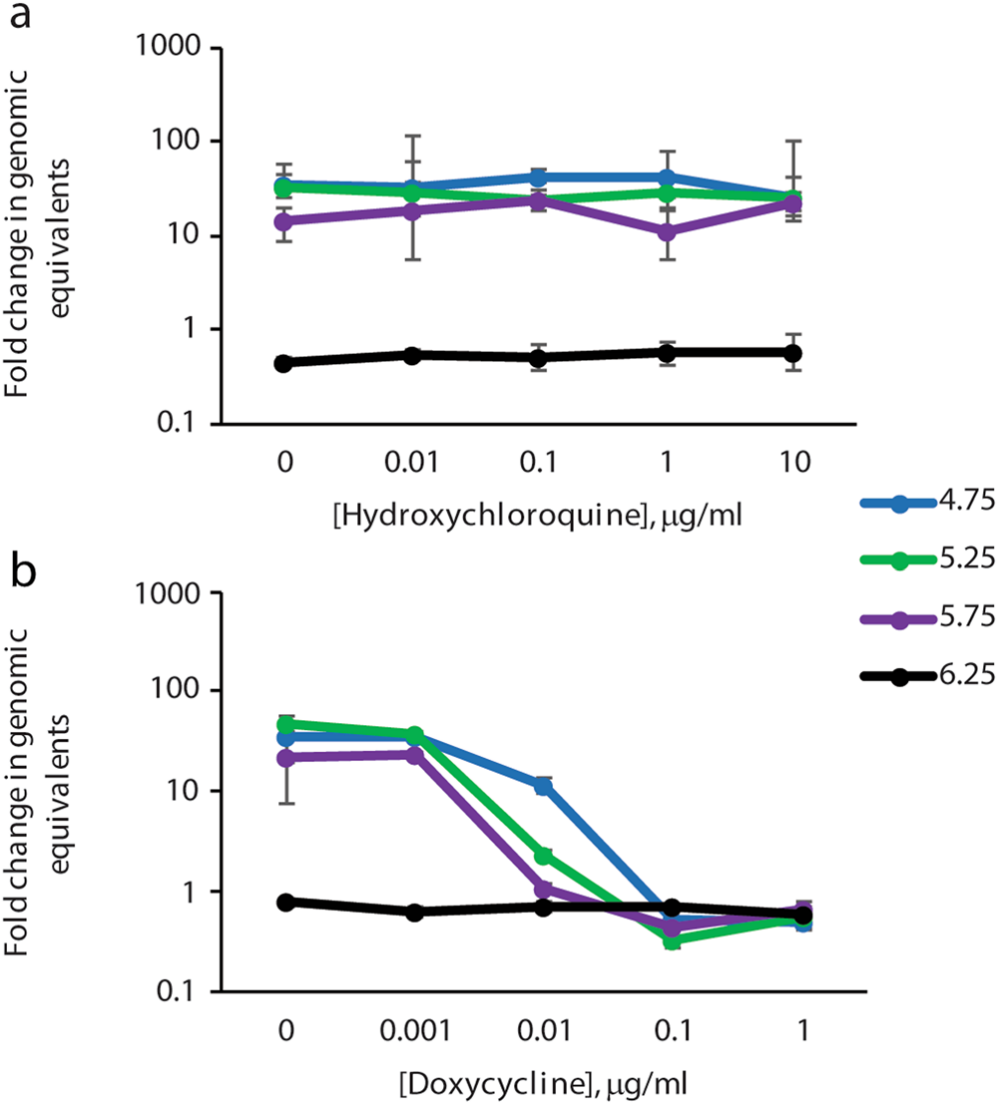
Impact of hydroxychloroquine on *C. burnetii* growth at different pH. In (**a**), *C. burnetii* were exposed to the indicated concentrations of hydroxychloroquine and no effect on growth was observed. In (**b**), hydroxychloroquine was added at 10 μg/ml to all of the cultures and doxycycline was titrated. The ratios of the means of genomes at day 7 to the means of genomes at the beginning of the culture were calculated to determine fold change in genomic equivalents. The standard errors of the means of genome equivalents were calculated and used to determine the upper and lower bounds of the ratios (fold change). The error bars depict the upper and lower bounds of fold change for each condition.

Combination therapy with doxycycline and hydroxychloroquine is recommended for patients with chronic Q fever^10^. Hydroxychloroquine is known to raise the pH of vacuoles that support *C. burnetii* replication by facilitating proton leakage out of these vacuoles^21^. We tested the effect of hydroxychloroquine on *C. burnetii* growth and survival in the ACCM-2 system at various buffered pHs. Hydroxychloroquine had no effect on growth of *C. burnetii* in this system regardless of the pH of the media (Fig. 4), suggesting that without the ability to impact intravacuolar pH, hydroxychloroquine is ineffective. As expected, no changes were observed in the pH of ACCM-2 media after the addition of hydroxychloroquine. This was evaluated by preparing aliquots of ACCM-2 media at a pH of 4.75, 5.25, 5.75, and 6.25 followed by addition of hydroxychloroquine to each aliquot at a concentration of 10 μg/ml. The pH of the media did not change after addition of the hydroxychloroquine. Treatment of cultures with varying doses of doxycycline with a fixed dose of 10 μg/ml hydroxychloroquine resulted in growth inhibition very similar to use of doxycycline alone (Fig. 4). Evaluation of the viability of NMP2 after treatment with hydroxychloroquine alone or in combination with doxycycline demonstrated that hydroxychloroquine had no impact on *C. burnetii* viability nor did it change the bacteriostatic activity of doxycycline in ACCM-2 (Table 3).

**Table 3:** Viability of *C. burnetii* after one week culture in ACCM-2 with a titration of hydroxychloroquine or a fixed amount of hydroxychloroquine plus a titration of doxycycline.

pH	[hydroxychloroquine], μg/ml				
	0	0.01	0.1	1	10
4.75	+	+	+	+	+
5.25	+	+	+	+	+
5.75	+	+	+	+	+
6.25	+	+	+	+	+
	**[doxycycline], μg/ml (with 10 μg/ml hydroxychloroquine)**				
**pH**	**0**	**0.001**	**0.01**	**0.1**	**1**
4.75	+	+	+	+	+
5.25	+	+	+	+	+
5.75	+	+	+	+	+
6.25	+	+	+	+	+

A “+” indicates growth on ACCM-2 plates after dilution from the treated culture. A “−“ indicates no growth.

## Discussion

The data presented here demonstrate the establishment of an axenic system for evaluating the impact of antimicrobial agents on *C. burnetii*. In this system, only actively replicating *C. burnetii* (LCV) are challenged with antibiotics. This system does not address the impact of these drugs on the SCV form of *C. burnetii*. However, it is expected that most antibiotics would have little efficacy on quiescent SCV due to the targeting of pathways involved in replication and/or protein synthesis. In the axenic system that was used in this study, erythromycin and azithromycin had very little effect on *C. burnetii* growth. It is unclear why the two macrolides tested did not have any efficacy in this system. There is limited data suggesting that some macrolides may have benefit for Q fever patients^11^, but in this *in vitro* system there was no effect on growth. It is possible that the macrolides have some benefit but do not inhibit *C. burnetii* directly, but also possible that the two tested here are not effective against *C. burnetii*.

It has been suggested that the benefit of hydroxychloroquine/doxycycline combination therapy is a result of the ability of hydroxychloroquine to raise the pH of vacuoles supporting replication of *C. burnetii* and that the raised pH of the vacuoles creates an environment where doxycycline and other antibiotics take on bactericidal activity^13,14^. The results here do demonstrate that there is some improvement of efficacy of doxycycline and moxifloxacin in ACCM-2 when pH is raised above 4.75, but this effect seems to only be a dose effect on bacteriostatic activity. In the ACCM-2 system, no bactericidal activity was observed for doxycycline or moxifloxacin. Rifampin did display bactericidal activity but this did not appear to be pH dependent. The pH of the media did not impact rifampin efficacy. Most antimicrobial agents will have some bactericidal activity if the concentration is high enough, but if the minimum bactericidal concentration is more than 4-fold greater than the MIC, then the drug is considered bacteriostatic^22^. In this study, bactericidal activity was not observed for doxycycline or moxifloxacin at concentrations 10–100 fold above the MIC regardless of pH, suggesting that these drugs should be considered bacteriostatic for *C. burnetii*. The bacteriostatic activity of doxycycline and bactericidal activity of rifampin are consistent with activities of these drugs on other bacteria^23,24^. Some bactericidal effects from fluoroquinolones such as moxifloxacin could be possible but were not observed against *C. burnetii* using the methods in this study.

The observation in previous studies that doxycycline efficacy is improved when infected cells are treated with hydroxychloroquine could be related to the transport of doxycycline across membranes. Tetracyclines and fluoroquinolones are thought to rely on pH gradients to move across cell membranes^25^. This means that doxycycline will more readily move from a lower pH to a higher pH. Because the intracellular pH of *C. burnetii* has been estimated to be 6–6.5 when the extracellular pH is around 4.75^26^, doxycycline should easily move from the low pH of the vacuole or ACCM-2 media to the cytoplasm of *C. burnetii*. However, in infected host cells, doxycycline will also need to pass from the host cell cytoplasm into the vacuole, and this transport could be inhibited by the lower pH of the vacuole compared to the cytoplasm. Addition of hydroxychloroquine could improve transport of the doxycycline into the vacuole *in vivo* and make more doxycycline available for movement into the *C. burnetii* cytoplasm. The pH of the *C. burnetii* cytoplasm should rise as the pH of the vacuole is increased, but the gradient will be reduced at higher extracellular pH, and this could reduce transport of doxycycline into the *C. burnetii* cytoplasm. Presumably there is a net gain in availability of doxycycline within *C. burnetii* when the pH of the vacuole is raised by hydroxychloroquine. Thus, addition of hydroxychloroquine *in vivo* could shift the dose curve of doxycycline by a mechanism that would not be observable in ACCM-2.

These data raise the possibility that the primary effect of hydroxychloroquine is to prevent *C. burnetii* replication by raising the pH on intracellular vacuoles to a level that does not support *C. burnetii* replication. In this scenario, doxycycline and hydroxychloroquine both act as bacteriostatic agents by different mechanisms- doxycycline by inhibition of protein synthesis^23^ and hydroxychloroquine by raising pH. The benefits of combination therapy have been clearly demonstrated^12^, but may not be the result of imparting bactericidal activity on doxycycline. Bactericidal activity by protein synthesis inhibitors generally requires that the bacteria be actively replicating, and the presence of a bacteriostatic agent can antagonize bactericidal activity of another agent^27^. The effectiveness of combination therapy suggests that this is not the result of bactericidal and bacteriostatic functions working at the same time.

The bactericidal activity against *C. burnetii* by rifampin in this study suggests that further study of this drug as an alternative therapy for Q fever is warranted. It is not clear how well results generated in this axenic system will translate to an intracellular *in vivo* reality, but the utility of rifampin against Q fever in clinical cases has been noted^28^. For patients that cannot tolerate long-term doxycycline therapy for chronic Q fever, effective alternatives are needed. These results suggest that rifampin is an alternative that should be explored.

## Methods

### Bacterial and antibiotic stocks

The *Coxiella burnetii* strain used in this study was Nine Mile Phase 2, clone 4 (NMP2), a low virulence strain that has a large genomic deletion that removes genes responsible for the complexity of LPS side chains^29^. Stocks of NMP2 were prepared by growth in ACCM-2 media at pH 4.75, and freezing in sodium phosphate glutamate (SPG) buffer. Antibiotics were purchased as follows: doxycycline hyclate, ciprofloxacin, rifampin, hydroxychloroquine, erythromycin, and levofloxacin (Sigma, St. Louis, MO), azithromycin dihydrate, and moxifloxacin hydrochloride (United States Pharmocopeia, Rockville, MD). Doxycycline hyclate, moxifloxacin hydrochloride, ciprofloxacin, and hydroxychloroquine stocks were prepared by dissolving in deionized water at a concentration of 1280 μg/ml. Levofloxacin was dissolved in an aqueous solution of 0.05 M NaOH at 1280 μg/ml. Erythromycin and azithromycin were dissolved in 95% ethanol at 1280 μg/ml.

### pH dependence of *C. burnetii* growth

To evaluate the pH dependence of *C. burnetii* grown in ACCM-2, media was prepared as described^17^ and pH adjusted using 1 N NaOH. T-25 flasks containing 7 mls of ACCM-2 at pH 4.5, 4.75, 5.0. 5.5, 6.0, 6.5, 7.0, and 7.5 were individually inoculated with 100 NMP2 organisms taken from frozen stocks and incubated for 8 days at 37 °C, 5% CO_2_ and 2.5% O_2_. Genome equivalents were determined by quantitative PCR (qPCR) from 200 μl aliquots taken on days 0 and 8.

### Quantitative PCR

Total genomic DNA was extracted from 200 μl samples using the QIAamp DNA mini kit tissue protocol (Qiagen, Valencia, CA) according to the manufacturer’s instructions. Genomic equivalents of *C. burnetii* in the sample were determined by performing qPCR targeting the *com1* gene as previously described^30^.

### Antibiotic susceptibility screening

Cultures of NMP2 were inoculated with approximately 1 × 10^7^ NMP2 per ml taken from frozen stock and grown for 8 days in ACCM-2 at pH 4.75, 37 °C, 5% CO_2_ and 2.5% O_2_. Antibiotics were added at days 0 and day 4 in sufficient quantity on both days to achieve a concentration of 10 μg/ml. This concentration was higher than the peak serum concentration (Cmax) observed in patients taking standard doses for all antibiotics except rifampin. Because of its higher Cmax, rifampin was added at 20 μg/ml. A 200 μl aliquot was taken from each culture immediately and again after 8 days incubation. Samples were analysed by qPCR and the fold change between days 0 and 8 calculated. Data presented are from two independent replicates.

### Antibiotic efficacy at variable pH

Four days prior to initiation of antibiotic treatment, 20 ml of ACCM-2 at pH 4.75 was mixed in a T75 flask with 1 × 10^8^ genome equivalents of NMP2 taken from frozen stock. The flask was incubated at 37 °C, 2.5% O_2,_ and 5% CO_2_ for four days, allowing the *C. burnetii* to transition from the small cell variant to the large cell variant and enter the log phase of replication. After four days of growth, 200 μl of culture was transferred into T25 flasks containing 7 ml of fresh ACCM-2 at 4.75, 5.25, 5.75, or 6.25 pH in duplicate. Antibiotics were added on days 1 and day 4 in sufficient quantity on both days to achieve concentrations of 0.001. 0.01. 0.1, 1, and 10 μg/ml. A 200 μl aliquot was taken from each sample on days 0 and 7 for quantification of *C. burnetii* by qPCR. The minimum inhibitory concentration (MIC) was defined as the lowest concentration tested that resulted in less than half of the quantity of *C. burnetii* compared to untreated controls. On day 7, an additional aliquot of 10 μl from each culture was then transferred to an ACCM-2 agar plate at pH 4.75. The plates were incubated for two weeks at 37 °C, 2.5% O_2,_ and 5% CO_2_, and colony growth was noted to determine if the cultures were viable. Although any detectable colonies on the plate would be reported as viable (+), all viable cultures had colonies too numerous to count. Separately, pH of ACCM-2 prepared at pH 4.5, 5.25, 5.75, and 6.25 was measured before and after the addition of doxycycline, moxifloxacin, rifampin, and hydroxychloroquine at 10 μg/ml to confirm that the antibiotics did not affect the pH of the cultures.

### Statistical analysis

The means and standard errors of the mean (SEM) of the quantity of *C. burnetii* were determined for the samples taken at day 1 and day 7. The ratios of day 7 to day 1 samples were then calculated to determine fold change. Using the means plus/minus SEM, the upper and lower bounds for fold change were calculated and plotted on the graphs. The 95% confidence intervals for Fig. 1 and Table 1 were calculated using GraphPad QuickCalcs.

### Disclaimer

The findings and conclusions in this report are those of the authors and do not necessarily represent the views of the C.D.C.

## Data Availability

The data generated and analyzed for the current study are available from the corresponding author on reasonable request.
